# Advances in AAV-mediated gene replacement therapy for pediatric monogenic neurological disorders

**DOI:** 10.1016/j.omtm.2024.101357

**Published:** 2024-10-16

**Authors:** Livia Zhou, Yafeng Wang, Yiran Xu, Yaodong Zhang, Changlian Zhu

**Affiliations:** 1 Henan Neurodevelopment Engineering Research Center for Children, Children’s Hospital Affiliated to Zhengzhou University, Henan Children’s Hospital Zhengzhou Children’s Hospital, Zhengzhou 450018, China; 2 Henan Key Laboratory of Child Brain Injury and Henan Pediatric Clinical Research Center, Institute of Neuroscience and The Third Affiliated Hospital of Zhengzhou University, Zhengzhou, Henan Province, China; 3 Center for Brain Repair and Rehabilitation, Institute of Neuroscience and Physiology, University of Gothenburg, Gothenburg, Sweden

**Keywords:** AAV, gene therapy, monogenetic diseases, pediatrics, neurology

## Abstract

Pediatric monogenetic diseases encompass a spectrum of debilitating neurological disorders that affect infants and children, often resulting in profound cognitive and motor impairments. Gene replacement therapy holds immense promise in addressing the underlying genetic defects responsible for these conditions. Adeno-associated virus (AAV) vectors have emerged as a leading platform for delivering therapeutic genes due to their safety profile and ability to transduce various cell types, including neurons. This review highlights recent advancements in AAV-mediated gene replacement therapy for pediatric monogenetic diseases, focusing on key preclinical and clinical studies. We discuss various strategies to enhance transduction efficiency, target specificity, and safety. Furthermore, we explore challenges such as immune responses, along with innovative approaches to overcome these obstacles. Moreover, we examine the clinical outcomes and safety profiles of AAV-based gene therapies in pediatric patients, providing insights into the feasibility and efficacy of these interventions. Finally, we discuss future directions and potential avenues for further research to optimize the therapeutic potential of AAV-delivered gene replacement therapy for pediatric encephalopathies, ultimately aiming to improve the quality of life for affected individuals and their families.

## Graphical abstract

**Figure:**
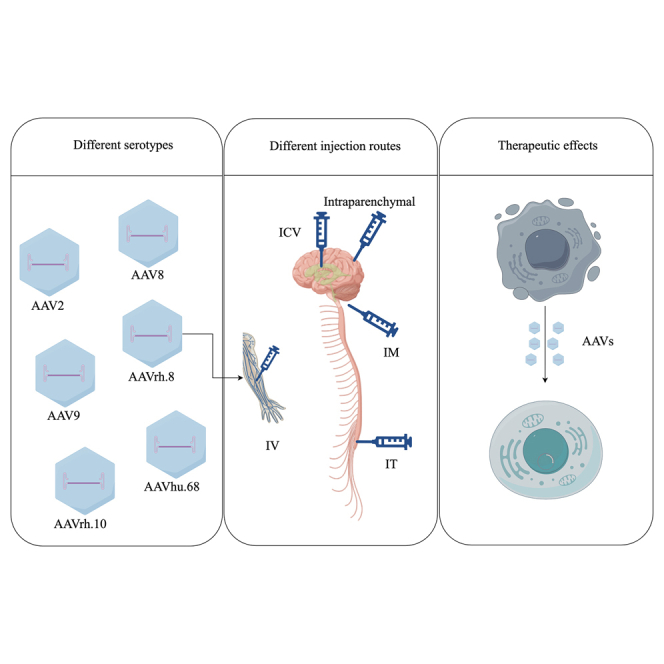

## Teaser

Zhu and colleagues review the promise of AAV-mediated gene replacement therapy for pediatric monogenic neurological disorders by addressing underlying genetic defects. Recent advancements in AAV vectors, particularly in enhancing safety, transduction efficiency, and targeting specificity, highlight the potential for improving the quality of life for affected children.

## Introduction

Rare diseases are defined as diseases affecting fewer than 200,000 patients in the US or less than 0.5‰ of the population by European standards. Approximately 80% of these diseases are monogenic, with 70% leading to neurological dysfunctions.^1^ The scarcity of effective treatments is largely attributable to restricted commercial capacity due to the limited patient population. However, recent advancements in gene therapy delivery vehicles and gene editing tools have ignited a beacon of hope for these rare monogenic diseases. Gene replacement therapy, for instance, offers a direct solution for monogenic recessive diseases resulting from loss-of-function mutations in critical genes, such as in Tay-Sachs disease and Gaucher disease. Conversely, gene editing tools provide a versatile approach, particularly apt for diseases characterized by gain-of-function mutations or a combination of loss- and gain-of-function mutations, such as Huntington’s disease.^2^ These innovative therapeutic strategies hold promise for revolutionizing the management of rare monogenic diseases.

Gene replacement therapy, a promising therapeutic approach for monogenic diseases, involves introducing a healthy copy of the gene to compensate for a mutant or dysfunctional one. This method, delivered via a suitable system, ensures the production of functional proteins. AAVs have emerged as the most promising delivery vectors for neurological diseases due to their demonstrated safety, ability to cross the blood-brain barrier (BBB), tropism for the central nervous system (CNS) with certain serotypes, and potential for engineering to enhance CNS tropism.^3^^,^^4^ Several AAV-based gene therapies have gained regulatory approval, each characterized by different injection routes, AAV serotypes, and disease targets. For instance, Luxturna, an AAV2-based gene therapy, was approved by the US Food and Drug Administration (FDA) for the treatment of *RPE65* mutation-associated retinal dystrophy following subretinal injection. Zolgensma, an AAV9-based therapy, received FDA approval for the treatment of spinal muscular atrophy induced by *SMN1* mutations after intravenous (i.v.) injection. Upstaza, another AAV2-based therapy, was approved by the European Medicines Agency for severe aromatic L-amino acid decarboxylase (AADC) deficiency following intraparenchymal injection. In addition, Elevidys, an AAVrh74-based therapy, was approved by the FDA for the treatment of Duchenne muscular dystrophy (DMD) resulting from mutations in the DMD gene, following i.v. administration.

AAV-based gene therapy for neurological diseases can be administered through various routes, each offering distinct advantages and targeting capabilities.^5^ i.v. injection allows for widespread distribution across the CNS and is particularly effective in reaching peripheral tissues, making it suitable for systemic conditions. In contrast, intra-cerebrospinal fluid (CSF) administration, including intrathecal (i.t.) and intracisternal injections, directly targets the CNS, enhancing gene delivery to the brain and spinal cord while minimizing peripheral exposure. Intraparenchymal injection, which involves direct delivery into specific brain regions, provides precise targeting of localized areas, making it ideal for focal neurological conditions. The choice of administration route is critical in therapeutic design, as it influences the distribution, efficacy, and safety profile of the gene therapy.^5^

**Figure 1:**
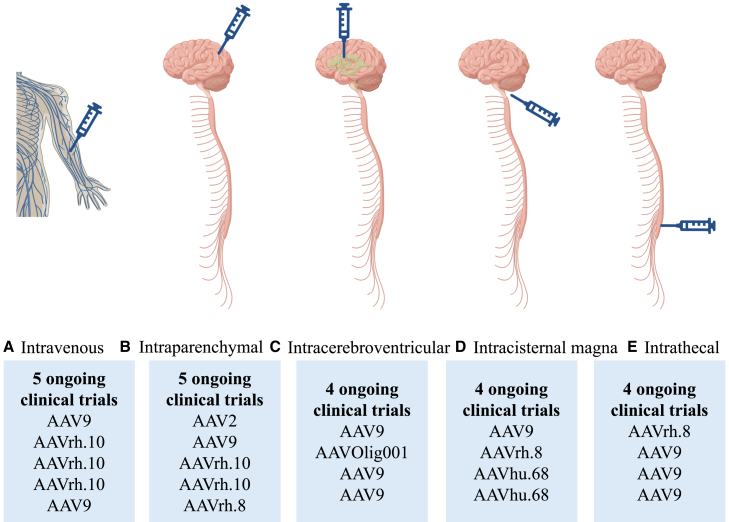
Routes of administration utilizing in ongoing clinical trials for AAV-based gene replacement therapy for pediatric and AAV serotypes unitizing in each route (A) Intravenous administration of AAV9 and AAVrh.10 in different ongoing clinical trials. (B) Intraparenchymal administration of AAV2, AAV9, AAVrh.10, and AAVrh.8 in different ongoing clinical trials. (C) Intracerebroventricular administration of AAV9 and AAVOlig001 in different ongoing clinical trials. (D) Intracisternal magna administration of AAV9, AAVrh.8, and AAVhu.68 in different ongoing clinical trials. (E) Intrathecal administration of AAVrh.8 and AAV9 in different ongoing clinical trials.

Given the underdevelopment of children’s immune systems and BBB, it is implied that pediatric patients may necessitate distinctive strategies or responses compared with adults.^6^^,^^7^ In addition, numerous monogenic diseases with neuropathologies are initiated during childhood or even infancy, leading to premature death or enduring motor and cognitive dysfunctions, such as type II Gaucher disease.^8^ Therefore, early intervention is paramount to prevent irreversible CNS afflictions, such as for neuronal ceroid lipofuscinoses.^9^ This review concentrates on the application of AAV-based gene replacement therapies for pediatric monogenetic diseases, with a particular emphasis on treatments currently undergoing clinical trials (Table 1; Figure 1) and the critical preclinical studies that helped initiate the translation to the clinical investigations (Table 2). It will encompass the recent advancements in preclinical studies and clinical trials and will discuss the challenges encountered in translating novel gene therapies from bench to bedside for pediatric monogenetic diseases.

**Table 1:** Continuing clinical trials of AAV-based gene replacement therapies for pediatric encephalopathy

Clinical trial	Disease	Sponsor	Phase	AAV	Transgene	Patients no.	Age	Initiated day	Injection route	Dose (vg)
NCT04903288	AADC deficiency	PTC Therapeutics	II	2	*AADC*	13	1–17 y	2021	intraputaminal	1.8E–11
NCT05765981	AADC deficiency	Shanghai Jiao Tong University	I	9	*AADC*	6	2–7 y	2023	intraputaminal	dose escalating
NCT03612869	MPSIIIA	Lysogene	II/III	rh.10	*SGSH*	20	≥6 m	2018	intraparenchymal	single dose
NCT01801709	MLD	Institut National de la Santé Et de la Recherche Médicale, France	I/II	rh.10	*ARSA*	5	6 m–4 y	2014	white matter	1.0 to 4.0E–12
NCT04411654	type II Gaucher disease	Prevail Therapeutics	I/II	9	*GBA1*	15	0–2 y	2021	i.c.m.	2-dose escalating
NCT06272149	type II Gaucher disease	Xinhua Hospital, Shanghai Jiao Tong University	I	9	*GBA1*	6	0–2 y	2023	i.c.v.	2-dose escalating
NCT04669535	GM2 gangliosidoses	University of Massachusetts Medical Health Center	I	rh.8	*HEXA/HEXB*	11	6 m–12 y	2021	intrathalamic and i.c.m./i.t.	4-dose escalating
NCT04798235	GM2 gangliosidoses	Taysha Gene Therapies	I/II	9	*HEXA/HEXB*	3	≤15 m	2021	i.t.	single dose
NCT03952637	GM1 ganglioside	National Human Genome Research Institute (NHGRI)	I/II	9	*GLB1*	45	6 m–12 y	2019	i.v.	1.5 to 4.5E–13/kg
NCT04713475	GM1 ganglioside	Passage Bio	I/II	hu.68	*GLB1*	26	1 m–2 y	2021	i.c.m.	3.3E–10 to 2.2E–11
NCT04693598	Krabbe disease	Forge Biologics	I/II	rh.10	*GALC*	6	0–1 y	2021	i.v.	2-dose escalating
NCT05739643	Krabbe disease	Forge Biologics	I/II	rh.10	*GALC*	9	children and adults	2023	i.v.	2-dose escalating
NCT06308718	Krabbe disease	Forge Biologics	?	rh.10	*GALC*	25	children and adults	2024	i.v.	single dose
NCT04771416	Krabbe disease	Passage Bio	I/II	hu.68	*GALC*	24	1–9 m	2022	i.c.m.	1.5E–11 to 5.0E–11
NCT04998396	Canavan disease	Aspa Therapeutics	I/II	9	*ASPA*	18	≤30 m	2021	i.v.	2-dose escalating
NCT04833907	Canavan disease	Myrtelle	I/II	Oligo001	*ASPA*	24	3–60 m	2021	i.c.v.	3.70E+13
NCT02362438	GAN	Taysha Gene	I	9	*GAN*	21	≥3 y	2015	i.t.	4-dose escalating
NCT06217861	GA-1	Vitalgen	I	9	*GCDH*	12	0–6 y	2024	i.c.v.	dose escalating
NCT06152237	Rett syndrome	Taysha Gene	I	9	MECP2	6	5–8 y	2023	i.t.	2-dose escalating
NCT05898620	Rett syndrome	Neurogene	I/II	9	MECP2	16	4–10 y	2023	i.c.v.	2-dose escalating

AADC, aromatic L-amino acid decarboxylase; MPS, mucopolysaccharidoses; MLD, metachromatic leukodystrophy; GAN, giant axonal neuropathy; GA-1, glutaric acidemia type 1; AAV, adeno-associated virus; i.c.m, intracisternal magna; y, years; m, months; i.c.v., intracerebroventricular; Ii.t, intrathecal; i.v., intravenous; vg, vector genomes.

**Table 2:** The critical preclinical studies that helped initiate the translation to clinical investigation

Disease	AAV	Transgene	Species	Injection route	Reference
LINCL	2	*CLN2*	mouse, rat, and NHPs	intraparenchymal	Passini et al.^10^; Sondhi et al.^11^
MLD	rh.10	*ARSA*	mouse and NHPs	intraparenchymal	Sevin et al.^12^; Cearley and Wolfe^13^; Piguet et al.^14^; Zerah et al.^15^
Sandhoff disease	rh.8	*HEXA* and *HEXB*	feline	intraparenchymal	Bradbury et al.^16^; McCurdy et al.^17^; McCurdy et al.^18^
GM1 gangliosidosis	9, rh.10, and Hu68	*GLB1*	mouse and feline	i.v. and i.c.m.	Liu et al.^19^; Hocquemiller et al.^14^; Hinderer et al.^20^
GLD	rh.10 and Hu68	*GALC*	canine, mouse, and NHPs	i.v., i.c.v., and i.c.m.	Bradbury et al.^21^; Rafi et al.^22^; Hordeaux et al.^23^
CD	9	*ASPA*	mouse	i.v.	Ahmed et al.^24^; Gessler et al.^25^
Rett syndrome	9	*Mecp2*	mouse	i.t.	Sinnett et al.^26^
DS	9	*SCN1A*	mouse and NHPs	i.c.v.	Andrew et al.^27^; Archana et al.^28^
GAN	9	*GAN*	mouse	i.t.	Bailey et al.^29^
GA-1	9	*GCDH*	mouse	i.c.v.	Guo et al.^30^

LINCL, late infantile neuronal ceroid lipofuscinoses; NHP, non-human primates; MLD, metachromatic leukodystrophy; i.v., intravenous; i.c.m., intracisternal magna; GLD, globoid cell leukodystrophy; i.c.v., intracerebroventricular; CD, Canavan disease; i.t., intrathecal; DS, Dravet syndrome; GAN, giant axonal neuropathy; GA-1, glutaric acidemia type 1.

## Diverse AAV variants transducing the brain

Due to the inherent challenges of achieving CNS transduction through systemic administration—especially the difficulty many vectors encounter in crossing the BBB—numerous AAV variants have been developed to enhance this capability. Accordingly, this section focuses on endogenous and engineered AAV capsids that have demonstrated effective CNS targeting when delivered intravenously.

AAV has been proven as a potent delivery vector to the brain. For example, AAV2 was the inaugural serotype widely utilized in CNS studies, particularly for intraparenchymal injections, with its efficacy substantiated both in approved therapeutic applications and numerous clinical trials.^31^ AAV9, however, has emerged as the most extensively tested serotype capable of crossing the BBB, demonstrating broad transduction of CNS cells, including astrocytes and neurons.^7^^,^^32^^,^^33^ Intriguingly, the cell types predominantly targeted by AAV9 post-i.v. administration may vary between adults and neonates. However, conflicting results have been reported; one study found that i.v. injection of AAV9 mainly targeted neurons in neonatal mice but astrocytes in adult mice,^7^ while another study reported twice as many neurons as astrocytes being transduced in adult mice following i.v. injection of AAV9.^34^ In addition, AAV9 can target a myriad of brain regions in neonatal mice, including the striatum, cortex, corpus callosum, hippocampus, midbrain, cerebellum, and others, with a preference for pyramidal and Purkinje neurons with long axons over GABAergic interneurons.^7^ While these effects are observed following i.v. injection, other administration routes, such as intra-CSF injection, also have the potential to target a broad range of brain regions.^35^

Subsequent research has identified novel serotypes, such as AAVrh.8 and AAVrh.10, which exhibit comparable or superior transduction of the CNS following systemic administration in adult rodents and non-human primates (NHPs).^36^ Moreover, AAVrh.10, AAVrh.39, AAVrh.43, AAV9, and AAV7 have demonstrated high transduction efficiency in both neurons and glial cells throughout the CNS in neonatal mice following facial vein injection.^37^ However, it should be noted that high systemic doses could compromise the immature neonatal BBB, thereby augmenting the transduction rate.^38^ This factor warrants caution when interpreting the capacity of specific AAVs to cross the neonatal or childhood BBB.

AAV-PHP.eB has been reported to exhibit broad brain area transduction following i.v. injection.^39^ However, this high transduction rate appears to be strain specific, with significant efficiency observed in the C57BL/6J mouse strain but not in other strains or species, including NHPs.^40^ Subsequently, new AAV capsids engineered from AAV-PHP.eB demonstrated enhanced CNS transduction efficiency following i.v. administration in both mice and NHPs.^41^ Recently, novel AAV9 variants, including AAV.CPP.16 and AAV.CPP.21, have shown superior capabilities in crossing the BBB and transducing the CNS, outperforming AAV9 after systemic administration, as demonstrated in various mouse models and NHPs.^42^ Similarly, AAV-MaCPNS1 and AAV-MaCPNS2, variants based on AAV9, have also displayed considerable improvements in CNS transduction compared with AAV9 following i.v. injection.^43^ However, these novel variants have yet to gain widespread acceptance, and their safety profiles remain underexplored. Therefore, comprehensive efficacy and safety studies are imperative before these variants progress to clinical trials.

The most clinically utilized serotype remains AAV9, followed by AAV2, AAVrh.10, and AAVhu.68. Beyond novel serotypes or variants, the development of self-complementary AAVs (scAAV) carrying double-stranded cDNA has resulted in higher and faster transduction rates, albeit at the expense of reduced loading capacity.^44^^,^^45^ Another approach to target CNS involves the modification of peripherally expressed enzymes to enhance their ability to cross the BBB.^46^^,^^47^^,^^48^ Furthermore, intranasal delivery has been developed as a method to administrate AAVs into the brain.^49^ However, these technologies are still in developmental stages and require further refinement before clinical application. A significant hurdle in utilizing AAVs to treat neurological diseases is the presence of pre-existing humoral immunity. For instance, the most frequently tested AAV serotypes in neuropathological diseases, AAV2 and AAV9, have a prevalence of 72% and 47% in the general population, respectively.^50^ This could significantly impede the use of systemically administered AAVs, potentially excluding approximately half of the patient population. Studies have also found that even low levels of pre-existing antibodies can dramatically hinder the efficacy of gene therapy.^51^^,^^52^ However, newborns or children who have fewer opportunities for exposure to natural AAVs, such as AAV9, may be less affected.^53^ Moreover, intraparenchymal, intracerebroventricular (i.c.v.), and i.t. injections are less affected by pre-existing immunity compared with i.v. injections due to the lower dose used and reduced systemic exposure of AAV.^54^^,^^55^^,^^56^^,^^57^^,^^58^

In conclusion, ongoing efforts in the development of novel CNS-targeting AAV variants, in conjunction with diverse administration routes, hold promise for advancing clinical applications. After delving into the diversity of AAVs in brain transduction and their advancements in novel serotypes, we now shift our focus to the specific applications of AAVs in current medical research and therapeutic practices. Particularly, we highlight the therapeutic potential and achievements of AAVs in targeting a range of specific diseases, including genetic disorders, neurodegenerative diseases, and lysosomal storage diseases. Continuous innovation and technological developments in the field of gene therapy provide new possibilities for addressing long-standing untreatable diseases.

## Lysosomal storage diseases

Lysosomal storage diseases (LSDs) are a group of inherited single-gene recessive disorders characterized by defects in lysosomal function due to mutations in critical enzymes. These defects lead to the accumulation of undigested substrates within cells, affecting both the CNS and peripheral organs. With over 70 different types identified, LSDs exhibit an incidence of 1 in 5,000 live births, with more than half manifesting neurological disorders.^59^^,^^60^ A distinguishing characteristic of LSDs is "cross-correction," wherein cells secreting deficient enzymes can be utilized by neighboring cells lacking or harboring mutated genes encoding these enzymes, rendering LSDs highly amenable to gene therapy interventions.^61^ This unique trait obviates the necessity for cell-specific targeting AAVs equipped with specific promoters, as any enzyme-expressing cell can effectively secrete the enzyme, which can then be taken up by neighboring cells. Notably, even enzyme concentrations as low as 1%–10% of normal levels can efficiently mitigate substrate accumulation.^62^ Furthermore, animal models faithfully recapitulate the clinical course of LSDs, underscoring the potential of gene therapy as a promising therapeutic avenue for these debilitating disorders.^62^^,^^63^

*Late infantile neuronal ceroid lipofuscinoses (LINCL)* is a devastating neurodegenerative disorder caused by mutations in the *CLN2* gene, resulting in a deficiency of palmitoyl protein thioesterase-1 (PPT1) and subsequent accumulation of autofluorescent lipopigments. Manifesting typically around age 18 months, LINCL is characterized by progressive visual and cognitive impairments, seizures, and premature mortality.^64^ Preclinical studies utilizing intracranial injection of AAV2-expressing PPT1 in rodent and NHP models demonstrated both efficacy and safety.^10^^,^^11^ Building upon these findings, a clinical trial involving 10 patients was conducted, wherein intraparenchymal injection of AAV2-*CLN2* at 12 brain locations with a total dose of 2.5E–12 vector genomes (vg) showed a gradual attenuation of neurological deficits, although adverse events, including fatal epilepticus in one patient and mild, transient humoral immune responses in four patients, were noted.^65^ Subsequent investigations using a lower dose of AAVrh.10h-*CLN2* exhibited a slower disease progression but demonstrated inferior efficacy compared with recombinant PPT1 therapy.^66^ Another investigation on i.t. administration of scAAV9-*CLN6* into less common subtype LICLN6 also holds promise, with a clinical trial completed in 2021 (NCT02725580). While these studies underscore the potential of AAV-*CLN2* or AAV-*CLN6* as a treatment modality for LINCL following intracranial or i.t. administration, respectively, further research is warranted to optimize dosing regimens and assess safety profiles, particularly considering alternative routes of administration.

*Mucopolysaccharidoses (MPSs)* encompass a spectrum of metabolic disorders stemming from deficiencies in enzymes crucial for glycosaminoglycan (GAG) breakdown. Notable instances include Hurler syndrome (MPS I), Hunter syndrome (MPS II), and Sanfilippo syndrome (MPS III), with symptoms often featuring developmental delays, cognitive impairment, skeletal anomalies, coarse facial traits, and organomegaly. MPS III notably targets the brain, highlighting its potential for gene therapy interventions aimed at this organ.^67^

*Mucopolysaccharidosis type IIIA (MPSIIIA)*, a rare lysosomal storage disorder attributable to mutations in the sulfamidase (*SGSH*) gene, presents with lysosomal heparan sulfate (HS) accumulation, resulting predominantly in neurodegeneration and peripheral impairments typically identifiable at age 3–4 years, culminating in adolescence fatality.^68^^,^^69^^,^^70^ In preclinical models, i.c.v. administration of AAV5 carrying *SGSH* alongside an enhancer demonstrated HS reduction, mitigated neuroinflammation, and ameliorated motor and cognitive functions.^71^ Similarly, i.v. delivery of AAV9-*SGSH,* targeting both central and peripheral organs, exhibited substantial reductions in GAG storage and neuroinflammation, ultimately prolonging the lifespan of MPSIIIA mouse models.^72^

In a phase I/II trial published in 2014, intracranial injections of 7.2E–11 vg/patient of AAVrh.10-*SGSH-IRES-SUMF1* (sulfatase-modifying factor) vector were simultaneously administered into 12 locations within the basal ganglia white matter of 4 children aged 2–6 years, all of whom exhibited cognitive decline despite retained ambulation. Throughout the study, no adverse events were observed, indicating the safety of the AAV products, procedural methodology, and immune suppressive regimen. MRI assessments revealed stable brain atrophy in two patients, while two others exhibited progressive atrophy. Remarkably, the youngest participant, aged 2 years and 8 months at the time of treatment, demonstrated a positive cognitive function recovery post-treatment. Although the efficacy data remain limited due to the small sample size, the favorable safety profile and observed cognitive improvements suggest the potential utility of intracranial injections in addressing MPSIIIA.^73^ These promising outcomes prompted the initiation of a phase II/III clinical investigation in 2018 (NCT03612869), although no published data are currently available. A separate clinical trial employing i.v. administration of scAAV9-*SGSH* at a dose of 3E–13 vg/kg was terminated due to insufficient efficacy (NCT04088734). An alternative approach using liver-targeted BBB penetration modified *SGSH*, offering a novel avenue for peripheral administration of gene therapies to treat neurological disorders,^48^ no clinical trials have yet been conducted to evaluate this strategy. These studies underscore the promising potential of using AAV-based gene therapies for MPSIIIA, although further confirmation through larger clinical studies is warranted to elucidate optimal administration routes and efficacy.

*Mucopolysaccharidosis type IIIB (MPSIIIB)*, characterized by deficiency of α-N-acetylglucosaminidase (NAGLU) and subsequent accumulation of HS, manifests cognitive decline typically appearing between age 2 and 4 years, predominantly affecting the CNS but also impacting somatic organs and leading to premature mortality. Patients typically exhibit no symptoms at birth but develop progressive neurological deficits culminating in premature death.^74^ A phase I/II study conducted by uniQure Biopharma in four MPSIIIB patients aged 20, 26, 30, and 53 months utilized AAV5 intracranial injection targeting multiple brain regions via 16 injection sites with concomitant immune suppression.^75^ Positive cognitive improvements were observed across all patients, albeit with six severe adverse events among 117 reported adverse events, necessitating further safety and efficacy evaluations. Another clinical trial using AAV9-*NAGLU* with a cytomegalovirus (CMV) promoter was terminated due to a lack of drug supply and commercial considerations (NCT03315182). These studies underscore the need for continued exploration of AAV-based gene replacement therapies for MPSIIIB.

*Metachromatic leukodystrophy (MLD)* represents a lysosomal storage disorder stemming from mutations within the *ARSA* gene, which encodes the arylsulfatase A enzyme. In MLD pathology, the deficient ASA function triggers sulfatide accumulation within lysosomes, predominantly affecting oligodendrocytes in the nervous system, consequently precipitating oligodendrocyte degeneration and demyelination. This aberrant sulfatide buildup disrupts the integrity of the myelin sheath, culminating in progressive neurological deterioration.^76^^,^^77^ Clinical manifestations of MLD encompass developmental regression, motor skill impairment, muscular weakness, seizures, and ultimately profound disability and premature mortality.^77^^,^^78^ Therapeutic modalities for MLD remain constrained, with supportive care primarily aimed at symptom management and enhancing quality of life.^76^ The onset age of MLD varies depending on the disease subtype.^79^^,^^80^ For example, late-infantile MLD is the most common form, typically presenting between age 1 and 2 years, with onset possible as early as 6 months or as late as 4 years; juvenile MLD usually occurs between age 3 and 10 years but may manifest as late as adolescence or early adulthood. Understanding the age-specific patterns of symptom onset aids in timely diagnosis and management strategies for individuals affected by MLD.

Intrastriatal administration of AAVrh10-*ARSA* exhibited superior efficacy compared with AAV5-*ARSA* in mitigating brain pathology in a murine model of MLD.^12^^,^^13^^,^^14^ Subsequent toxicological assessment following intrastriatal delivery of AAVrh10-*ARSA* demonstrated an absence of severe adverse events, with discernible neuroinflammatory responses observed solely in the high-dose group, thereby advocating for further clinical exploration of intrastriatal injection of AAVrh10-*ARSA*.^15^ In a clinical trial (NCT01801709) initiated in 2014, 5 patients aged between 6 months and 4 years were enrolled, receiving 12 simultaneous injections of AAVrh10-*ARSA* into the white matter; specifically, 2 patients received a low dose totaling 1E–12 vg, while 3 patients received a high dose totaling 4E–12 vg. However, clinical data from this trial remains undisclosed. Thus, AAV-*ARSA* holds promise for treating MLD; however, additional administration routes may warrant further investigation to facilitate its clinical use. In addition, it is noteworthy that the *ex vivo* gene therapy product Lenmeldy received FDA approval in March 2024.

*Type II Gaucher disease* also referred to as acute neuronopathic Gaucher disease, represents a rare and severe manifestation of Gaucher disease. Typically, symptoms emerge early in infancy, often within the first few months of life, and are characterized by profound neurological complications, including progressive brain damage, seizures, developmental regression, muscle rigidity, and respiratory distress.^8^^,^^81^ The disease follows a rapid progression, and affected individuals frequently experience a markedly shortened lifespan, seldom surviving beyond early childhood.^8^^,^^82^ The swift onset and severity of neurological manifestations in type II Gaucher disease pose significant challenges for enzyme replacement therapy (ERT) to yield substantive therapeutic benefits. By the time symptoms become apparent, considerable neurological damage may have already ensued, thereby limiting the efficacy of ERT in mitigating or arresting disease progression.^83^ Notably, clinical investigations are underway to explore alternative therapeutic modalities, including a phase I/II trial initiated in the US in 2020 (NCT04411654) employing intracisternal magna (i.c.m.) delivery of AAV9 carrying the glucocerebrosidase gene (*GBA1*) to infants, as well as a phase I trial in China initiated in 2023 (NCT06272149) utilizing i.c.v. injection of AAV9-*GBA1*; however, outcomes from these trials remain pending.

*GM2 gangliosidoses* are hereditary lysosomal storage disorders attributed to mutations in the *GM2A* genes, subsequently fostering the accumulation of GM2 ganglioside within neuronal cells. This accumulation instigates progressive neurodegeneration and instills severe neurological symptoms, such as developmental regression, seizures, and motor function impairment.^84^ One of the main forms of GM2 gangliosidoses is Tay-Sachs disease associated with mutations in the *HEXA* gene, which encodes the α subunit of the lysosomal enzyme β-hexosaminidase A (HEXA), another form of GM2 gangliosidosis, known as Sandhoff disease, is specifically linked to mutations in the *HEXB* gene. The *HEXB* gene codes for the β subunit of HEXA as well as β-hexosaminidase B (HEXB).^85^ Currently, curative interventions for GM2 gangliosidoses are unavailable, with management predominantly reliant on supportive care.^86^

In pursuit of optimal therapeutic efficacy, studies have underscored the necessity of co-expressing both HEXA and HEXB.^87^^,^^88^ Bilateral injection of AAV1 carrying *HEXA* and *HEXB* into the deep cerebellar nuclei (DCN) and thalamus has significantly prolonged the lifespan of Sandhoff disease mouse and feline models.^16^^,^^89^ Subsequently, AAVrh8 vectors were employed to co-deliver *HEXA* and *HEXB* in a 1:1 ratio into the thalamus and DCN, yielding prolonged survival in a Sandhoff disease feline model.^16^^,^^17^^,^^18^ Further investigations utilizing i.c.v. injection of AAVrh8-*HEXA* and AAVrh8-*HEXB* have also shown promising outcomes.^90^ These compelling preclinical findings have paved the way for clinical trials.

A single clinical trial enrolled two patients, wherein one subject underwent i.t. and intrathalamic administration at age 7 months, while the other received i.t. and i.c.m. injections at age 30 months. Encouragingly, both individuals exhibited favorable safety profiles following treatment, heightened CSF HEXA activity levels, and an extended period of seizure remission.^91^ One ongoing clinical study initiated in 2021 involves the bilateral co-administration of AAVrh.8-*HEXA* and AAVrh.8-*HEXB* in a 1:1 ratio into the thalamus and dual i.c.m./i.t. administration into the CSF, although results are pending disclosure (NCT04669535). Subsequently, a dose-escalation study targeting infants is currently underway.^31^ Another clinical trial employing a distinct strategy involves packaging both *HEXA* and *HEXB* into a single vector, AAV9-*HEXB-P2A-HEXA*, for IT administration, with data yet to be disclosed (NCT04798235). These clinical endeavors hold promise for advancing therapeutic interventions for GM2-gangliosidoses.

*GM1 gangliosidosis*, a rare and yet profoundly debilitating genetic ailment, manifests through the intracellular buildup of GM1 ganglioside across diverse bodily tissues. Stemming from mutations within the *GLB1* gene, the disorder instigates a deficiency in β-galactosidase activity, impeding the proper catabolism of GM1 ganglioside and fostering its progressive accumulation. This aberrant lipid metabolism precipitates escalating neurodegenerative and systemic sequelae.^92^ The clinical presentation of GM1 gangliosidosis varies widely depending on the subtype and severity of the disease. The three main subtypes include infantile, late infantile/juvenile, and adult forms, each with a distinct age of onset, symptoms, and prognosis.^93^

Various injection routes and AAV serotypes have been explored across different models of GM1 gangliosidosis.^94^ For instance, i.v. administration of AAV9-*GLB1* in a mouse model exhibited encouraging outcomes.^19^ Similarly, in mouse and cat models, AAVrh10-*GLB1* delivered via CSF demonstrated extensive brain transduction, particularly with i.c.v. and i.c.m. injections, with subsequent NHP studies indicating widespread β-galactosidase expression in the brain and an absence of severe adverse effects.^95^ Furthermore, i.c.v. injections of AAVhu68-*GLB1* in another study successfully mitigated neurological deficits and motor dysfunction, thereby extending the survival.^20^ These promising findings across diverse AAV serotypes and disease models have prompted the initiation of clinical trials.

The clinical investigation involving i.v. administration of AAV9-*GLB1* in both infant and juvenile GM1 gangliosidosis patients is currently underway, with no specified date for completion (NCT03952637). Regrettably, the clinical trial employing i.c.m. injection of AAVrh10-*GLB1* in infants was halted due to the discontinuation of the sponsoring company, unrelated to safety concerns (NCT04273269). Concurrently, the study evaluating i.c.m. injection of AAVhu68-*GLB1* in infant and juvenile GM1 gangliosidosis patients is ongoing, with outcomes yet to be disclosed (NCT04713475).

*Krabbe disease*, or globoid cell leukodystrophy (GLD), represents a rare and severe genetic disorder marked by the deficiency of galactocerebrosidase enzyme activity, leading to the accumulation of detrimental compounds such as galactosylceramide and psychosine within the nervous system. This accumulation precipitates progressive damage to the myelin sheath encompassing nerve cells, resulting in neurological deterioration and systemic complications.^96^^,^^97^ Late-infantile- and juvenile-onset variants of Krabbe disease typically emerge in childhood, typically ranging from age 6 months to 10 years, presenting with symptoms encompassing developmental regression, loss of motor function, muscular rigidity, visual impairment, and cognitive decline. While the disease progression in late-infantile- and juvenile-onset forms tends to be slower compared with the infantile counterpart, affected individuals still confront considerable challenges and may experience a shortened lifespan.^98^

A combination of i.v. and i.c.v. administration of AAVrh.10-*GALC* demonstrated promising outcomes, including extended lifespan and diminished neuropathology in a canine model of GLD.^21^ In addition, another investigation exhibited a more than 10-fold increase in lifespan following i.v. delivery of AAVrh.10-*GALC* combined with hematopoietic stem cell transplantation (HSCT) in a murine GLD model.^22^ These findings represent significant advancements in Krabbe disease treatment, prompting the initiation of several clinical trials. In 2021 and 2023, respectively, 2 clinical trials commenced, involving a total of 15 patients who received i.v. administration of AAVrh.10-*GALC* at either low or high doses after prior HSCT treatment, with data yet to be disclosed (NCT04693598 and NCT05739643). Furthermore, a larger study involving 25 patients is set to commence in 2024, aiming to evaluate the long-term safety of i.v. injection of AAVrh.10-*GALC* over a 36-month follow-up period (NCT06308718).

In mouse models of Krabbe disease, i.c.v. injection of AAVHu68-*GALC* at a total dose of 1E–11 vg resulted in a more than 3-fold increase in life expectancy.^23^ Similarly, in a canine model of Krabbe disease, i.c.m. injection of AAVHu68-*GALC* at a total dose of 3E–13 vg led to a more than 7-fold extension in lifespan, accompanied by reductions in neuroinflammation and demyelination.^23^ Furthermore, a safety evaluation in NHPs following i.c.m. administration of AAVHu68-*GALC* revealed no severe adverse events. These findings have paved the way for subsequent clinical trials (NCT04771416), initiated in 2022, which involves i.c.m. injection of AAVHu68-*GALC* in 24 patients aged between 1 and 9 months, with two dosage options: 1.5E–11 or 5.0E–11 vg as a single dose. However, the study was suspended due to a change in the sponsor company’s strategy. Despite the encouraging therapeutic effects and favorable safety profile, a separate mouse study using AAV9-GALC indicated dysregulation of therapeutic GALC 6–8 months post-administration, potentially due to the exhaustion of the delivered therapeutic episomal DNA.^99^ This finding underscores the need for caution and long-term observation in clinical trials.

*Canavan disease (CD)* is a rare genetic disorder resulting from autosomal recessive mutations in the aspartoacylase (*ASPA*) gene, leading to deficient breakdown of N-acetylaspartic acid (NAA) within the brain. Elevated NAA levels can disrupt the myelination of white matter and precipitate spongiform neurodegeneration. The condition is typified by profound progressive neurological deficits, encompassing developmental delay, intellectual disability, hypotonia, macrocephaly, optic atrophy, and premature mortality.^100^^,^^101^

In CD mouse models, i.v. injections of AAV9, AAVrh.8, and AAVrh.10 carrying *ASPA* at various postnatal days (P0, P6, P13, and P20) exhibited promising outcomes, including normalization of NAA levels, enhanced myelination, rescue of retinopathy and neuropathy, and prolonged survival time. However, the efficacy of these treatments was found to be limited and not sustained.^24^ Subsequent optimization of these therapeutic approaches demonstrated promising results in both preventing and halting disease progression in CD mouse models.^25^ These encouraging preclinical findings have spurred the initiation of subsequent clinical trials, underscoring the potential for further advancements in CD treatment strategies.

Early clinical trials involving infants and children aged 4–83 months utilized recombinant AAV2-carrying *ASPA*, administered via multiple intracranial injection sites, resulting in some but limited reduction in NAA levels and prevention of brain deficits during a 5-year follow-up period.^102^^,^^103^ Building upon promising preclinical results, a clinical study initiated in 2021 (NCT04998396) employed i.v. administration of AAV9-*ASPA* in patients up to 30 months old; however, the data from this trial have yet to be disclosed. In pursuit of optimizing therapeutic efficacy, a clinical investigation of i.v. and i.c.v. administrations of AAV9-*ASPA* under the control of a CMV enhancer and a chicken β-actin (CB6) promoter targeted pediatric subjects aged 18–24 months (NCT05317780). Regrettably, the study is currently listed as “no longer available,” precluding access to any disclosed data or findings. Another study (NCT04833907) utilized i.c.v. injection of AAVOlig001-*ASPA* targeting oligodendrocytes in the brains of patients aged 3–60 months, with a total dose of 3.7E–13. The AAVOlig001 is a newly developed AAV capsid that can facilitate specific targeting of oligodendrocytes^104^; however, the results have not been disclosed. While current AAVs, such as AAV9, primarily transduce neurons and astrocytes,^7^ this study’s targeting of oligodendrocytes may offer a potentially safer and more direct approach for treating CD with oligodendrocyte degeneration.

Notably, while many LSDs exhibit neuropathology, some are not directly linked to gene deficiencies in the CNS. For instance, in phenylketonuria, neurocognitive impairment arises from the accumulation of phenylalanine (Phe) in the blood and brain due to the liver’s inability to metabolize Phe, necessitating treatment strategies targeting the liver.^105^ This highlights the importance of tailored approaches based on the specific pathophysiology of each disorder. Although systemic factors may not directly cause neuropathology, they can influence its progression, potentially through mechanisms such as inflammation or disruption of the BBB.

## Other monogenic neurological disorders

In addition to LSDs, several other monogenic disorders are associated with neurological dysfunction. For example, deficiencies in AADC and Rett syndrome are linked to developmental delays, while adrenoleukodystrophy (ALD) can result in vision impairment. Dravet syndrome (DS) predominantly manifests as seizures, and giant axonal neuropathy (GAN) is characterized by sensory dysfunction. Moreover, cognitive decline is observed in disorders such as Rett syndrome, ALD, and glutaric acidemia type 1 (GA-1). These conditions also contribute to motor dysfunction. Detailed descriptions of these disorders are provided below.

## AADC deficiency

AADC deficiency arises from mutations occurring within the *DDC* gene, responsible for encoding the AADC enzyme pivotal in the biosynthesis of neurotransmitters such as dopamine and serotonin. Dysfunction in AADC due to genetic mutations disrupts neurotransmitter synthesis, precipitating a spectrum of neurological and developmental manifestations commonly observed in infancy or early childhood.^106^ Clinical presentations of AADC deficiency encompass hypotonia, developmental delays, movement disorders including dystonia and chorea, oculogyric crises characterized by involuntary upward eye movements, autonomic dysregulation, and sleep disturbances. The severity of symptoms displays considerable interindividual variability, with a grim prognosis often characterized by fatality before the age of 5 years.^103^^,^^106^

Several clinical trials utilizing AAV2-*AADC* bilateral intraputaminal injection have demonstrated promising outcomes, including improvements in biomarkers and motor function. For instance, direct administration of 1 to 2E–11 AAV2-*AADC* vectors into the putamen yielded no severe adverse events attributable to the vectors, with treatment efficacy observed to be more pronounced in younger patients.^107^^,^^108^ The success of these trials has directly contributed to the approval of Upstaza by PTC Therapeutics for children aged 18 months and older in Europe and the UK. PTC Therapeutics has initiated another phase II clinical trial in the US, projected for completion in 2028 (NCT04903288). While a separate clinical trial employing AAV9-*AADC* has commenced in Shanghai, with an estimated completion in 2029 (NCT05765981). To enhance therapeutic outcomes and extend coverage to broader brain regions, a clinical study has explored intracranial injection of AAV2-*AADC* into the substantia nigra pars compacta (SNc) and ventral tegmental area (VTA), targeting dopaminergic neurons across nigrostriatal, mesolimbic, and mesocortical pathways. This approach has demonstrated both safety and significant improvements in motor and non-motor symptoms.^109^ However, considering the convenience of drug delivery, intraputaminal injection may be preferred over deeper SNc and VTA regions due to easier targeting and reduced injection risks, despite potentially slightly lower efficacy.

## Rett syndrome

Rett syndrome is a rare neurodevelopmental disorder primarily affecting females, typically manifesting between age 6 and 18 months. Rett syndrome is characterized by developmental regression, loss of acquired skills, and a range of neurological and physical impairments, and it poses significant challenges for affected individuals and their families. The condition is often marked by repetitive hand movements, impaired motor coordination, breathing irregularities, seizures, and intellectual disabilities.^110^ While most cases of Rett syndrome are caused by mutations in the *MECP2* gene, its exact pathophysiology remains complex and multifaceted.^111^ It is noteworthy that the regulation of the *MECP2* gene must be precise, as both overexpression and hypoexpression can be detrimental to brain function. Overexpression of the *MECP2* gene can lead to neurotoxicity and impair synaptic plasticity, while hypoexpression results in insufficient levels of the *MECP2* protein, contributing to the pathogenesis of Rett syndrome.^112^^,^^113^^,^^114^ This underscores the importance of understanding the intricate molecular mechanisms involved in maintaining optimal *MECP2* levels, offering valuable insights for the development of targeted therapeutic interventions for Rett syndrome.

A preclinical investigation employing truncated mini*MECP2* with a miRNA binding site demonstrated enhanced safety profiles without compromising efficacy in a Rett syndrome murine model.^26^ Another approach involved the utilization of an instability-prone *Mecp2* cassette to induce mRNA instability, thereby mitigating *Mecp2* overexpression in a Rett syndrome murine model.^115^ Encouraging safety and efficacy enhancements observed in these preclinical models prompted the initiation of clinical trials. One such trial involved the i.t. administration of AAV9-mini*MECP2* to patients aged 5–8 years (NCT06152237). Another trial employed i.c.v. delivery of AAV9-*MECP2* to patients aged 4–10 years, utilizing a technology called Expression Attenuation via Construct Tuning (EXACT) aimed at preventing *MECP2* overexpression (NCT05898620). However, no results have been disclosed for either study.

## ALD

ALD is a rare X-linked genetic disorder attributed to mutations in the *ABCD1* gene, which encodes the adrenoleukodystrophy protein (ALDP), crucial for peroxisome membrane integrity and very-long-chain fatty acids (VLCFA) trafficking. Dysfunctional ALDP impairs peroxisomal VLCFA metabolism, leading to their accumulation and subsequent progressive demyelination of the CNS and adrenal insufficiency.^116^ Childhood cerebral ALD (CCALD) represents the most severe and prevalent manifestation, predominantly afflicting boys aged 4–10 years.^117^ Characterized by swift neurological decline, including cognitive regression, behavioral alterations, vision impairment, and motor deficits, CCALD often culminates in profound disability or fatality within a short span post-symptom onset, underscoring the urgent need for intervention strategies.^118^ While current therapeutic approaches, including dietary VLCFA restriction and HSCT, offer some degree of disease progression attenuation, they remain non-curative and beset by limitations.^118^^,^^119^ Notably, *ex vivo* lentiviral gene therapies utilizing human ALDP-encoding constructs for blood stem cell transduction have shown promise, leading to FDA approval in 2022 following phase II clinical trials by Bluebird Bio.^120^ Nonetheless, the exorbitant cost of up to 3 million US dollars per treatment poses a significant accessibility challenge. Furthermore, ongoing phase III trials (NCT03852498) seek to further evaluate the efficacy and safety profile of this therapeutic modality.

AAV-based gene therapy emerges as a promising avenue for treating ALD by rectifying aberrant VLCFA metabolism and halting neurodegeneration. However, unlike in LSDs, ALDP is not a secretive protein, meaning the corrected protein cannot pass from one cell to another in ALD; thus, precisely delivering *ABCD1* to disease-related cell types is critical. Preclinical investigations revealed predominant ALDP expression in glia and endothelial cells, with limited presence in neurons.^121^^,^^122^ However, the exact mechanisms underlying axonal degeneration and white matter injury remain elusive. The absence of ALDP across multiple cell types likely contributes to disease progression.^123^^,^^124^^,^^125^ A comparative study utilizing i.v. and i.c.v. administration of AAV9-*ABCD1* with CMV enhancer/chicken β-actin hybrid promoter demonstrated the former’s broader brain and spinal cord distribution and effective reduction of brain VLCFA levels in *ABCD*−/− mice. Moreover, i.v. administration of AAV9-*ABCD1* could efficiently target the adrenal gland and potentially alleviate ALDP deficiency therein.^126^ Despite the higher expression levels observed with i.c.v. injection in the brain, the limited distribution within the brain possibly resulted in its failure to reduce VLCFA levels.^126^ Further exploration employing diverse cell-type-specific promoters for *ABCD1* gene delivery could elucidate the predominant cell types implicated in ALD pathology, facilitating the design of more targeted AAV vectors to mitigate the risk of off-target effects, such as neuronal toxicity, and to optimize therapeutic efficacy.

Although preclinical studies in ALD mouse models have shown the effectiveness of gene replacement therapy in reducing VLCFA levels, improving myelination, and extending survival,^126^ the translation of these findings into clinical benefits regarding dosing and timing remains unclear. Ongoing phase I/II clinical trials investigating the safety and efficacy of i.t. injection of AAV9-*ABCD1* for adrenomyeloneuropathy in adult patients since 2022 (NCT05394064) represent a significant step toward elucidating the therapeutic potential of AAV-based gene therapy in ALD. However, clinical trials for AAV-based gene therapies targeting CCALD are notably absent, possibly due to safety concerns and challenges related to efficient gene delivery to the brain. Early diagnosis is emphasized as a crucial factor for the successful application of AAV-based gene therapy in ALD, akin to other treatment modalities such as HSCT.^127^

## DS

DS is a rare and severe form of childhood epilepsy characterized by frequent seizures, cognitive impairment, and developmental delays.^128^^,^^129^ The majority of DS cases are caused by mutations in the *SCN1A* gene, which encodes the α subunit of the voltage-gated sodium channel Nav1.1. Loss-of-function mutations in *SCN1A* lead to hyperexcitability of neurons and increased susceptibility to seizures.^130^ Despite current treatment options, including antiepileptic drugs and a ketogenic diet, many patients with DS continue to experience debilitating seizures and cognitive impairments. Therefore, there is an urgent need for novel therapeutic approaches to address the underlying molecular pathology of DS.

AAV vectors represent attractive candidates for gene therapy owing to their efficient transduction of neurons, long-lasting gene expression, and favorable safety profile. However, the limited loading capacity of AAV poses challenges for delivering large genes such as those encoding sodium channel α subunits. Various strategies have been explored to target *SCN1A*-positive DS using AAV-based therapies. One such approach involves the use of a drug called ETX101, which utilizes AAV9 to deliver a GABAergic regulatory element and an engineered transcription factor to boost transcription of the endogenous *SCN1A* gene. Promising results in a preclinical mouse model included reduced hyperthermic seizures and mortality rates.^27^ Subsequent NHP studies confirmed the safety of i.c.v. injection of ETX101.^28^ Currently, three ongoing clinical trials, initiated or set to begin in 2024 (NCT06283212, NCT06112275, and NCT05419492), aim to further investigate this therapy. In addition, other AAV-delivered gene therapies, such as AAV-*SCN1B*, which enhances Nav1.1 expression in GABAergic neurons,^131^ as well as AAV-delivered gene editing therapies,^132^^,^^133^^,^^134^ have shown promising results in preclinical studies but have yet to progress to clinical trials.

## GAN

GAN is an autosomal recessive monogenetic disease. The mutation of the *GAN* gene leads to the loss of function of gigatons protein, which further leads to the enlarged axons filled with disorganized intermediate filaments (IFs) in multiple cell types, including neurons and glial cells in both the spinal cord and the brain.^135^^,^^136^^,^^137^^,^^138^ Children with this mutation usually have motor and sensory dysfunction and die in the second or third decade of life.^139^ Currently, there is no effective treatment for GAN.

AAV2-GAN, employing a CMV promoter, demonstrated the ability to reduce IF accumulation in human cell-cultured fibroblasts.^140^ In another *in vitro* study, lentivirus delivery of GAN reduced IF aggregates in motor neurons derived from induced pluripotent stem cells of GAN patients.^141^ In a GAN mouse model aged 19–20 months, i.c.m. injection of 10 μL of a total of 7.2 E–10 vg AAV9-*CMV-GAN* resulted in extensive transduction of large areas in the brainstem and spinal cord, leading to complete clearance of peripherin aggregates in these regions, albeit not in the brain.^140^ Moreover, unilateral brain injection of AAV9/JeT-*GAN* into the striatum and cortex reduced IF aggregation in the injected areas, although further functional assessments were not conducted in these mice, leaving the functional recovery and survival rate unknown.^29^ i.t. injection of 5E–10 vg of AAV9/JeT-*GAN* in the GAN mouse model delayed the onset of motor dysfunction and partly resolved later motor dysfunction, with long-term gigaxonin expression observed in the spinal cord and dorsal root ganglia.^29^ Based on these promising preclinical findings, a clinical study was initiated, involving i.t. injection of four escalating doses of scAAV9-*GAN* in children or adults aged 3 years and older, with results yet to be disclosed (NCT02362438).

## GA-1

GA-1 arises from mutations in the *GCDH* gene, culminating in the accumulation of glutaric acid (GA) and 3-hydroxy-glutaric acid (3-OH-GA) in the brain, ultimately resulting in brain injury and behavioral dysfunction.^142^ With approximately 75,000 affected individuals worldwide, typically with a life expectancy of 2–3 years, advancements in early diagnosis and appropriate care have led to improved outcomes, although 30% of survivors still endure long-term neurological dysfunction.^142^ In a GA-1 mouse model, i.c.v. delivery of scAAV9 carrying the *GCDH* gene with a dose of 1.5E–10 vg significantly increased survival rates.^30^ To further explore this potential therapy, a clinical trial was initiated in 2024, involving i.c.v. injection of recombinant AAV9-*GCDH* with escalating doses administered to patients under 6 years old (NCT06217861). However, as of now, the results of the clinical trial have not been disclosed.

## Challenges and future directions

Despite the promising preclinical and clinical data (Tables 1 and 2), several challenges remain in translating AAV-based gene replacement therapies into patients. These include optimizing delivery methods, ensuring targeted and sustained gene expression, addressing immune responses, and minimizing potential adverse effects. Future research efforts should focus on refining therapeutic strategies and developing combinatorial approaches to enhance therapeutic efficacy. Moreover, large-scale clinical trials are needed to establish the safety, efficacy, and long-term outcomes of gene therapy in these patients.

## Optimizing delivery methods

Certain diseases characterized by specific areas of brain degeneration can be targeted effectively through local injection, particularly when employing advanced techniques such as MRI-guided convection-enhanced delivery to enhance AAV distribution.^109^ Several monogenetic diseases affect both peripheral and CNS, with specific brain regions exhibiting degeneration, such as the white matter in Krabbe disease. Targeted administration routes like parenchymal or systemic injections often exhibit limited efficacy against these diseases. Thus, a combined delivery approach has been explored (Tables 1 and 2), leveraging both i.c.v. and i.v. routes to maximize therapeutic efficiency, as demonstrated in a clinical trial for CD (NCT05317780). Other studies have successfully combined i.t. injections with i.c.m. or intrathalamic injections for treating Tay-Sachs disease, confirming both safety and efficacy.^91^ An ongoing clinical trial also combined i.t. or i.c.m. with introthalamic injection (NCT04669535). Furthermore, the capacity of AAV to traverse the BBB significantly impacts the selection of administration routes, wherein less invasive approaches such as i.v. or IT routes could supplant the invasiveness associated with intraparenchymal or i.c.v. injections for AAV vectors exhibiting robust BBB penetration. Novel delivery routes, such as nasal delivery, are also under development.^49^ Consequently, it becomes imperative to strike a delicate balance between the efficacy and safety profiles of diverse administration routes. Combining local brain and systemic administration routes may yield optimal drug efficacy, albeit at a heightened safety risk. The advent of novel AAV capsids endowed with enhanced BBB-penetrating capabilities and novel administration routes hold promise for bolstering both safety and efficacy in future therapeutic interventions.

## AAV tropisms and loading capacity

AAV tropisms play a crucial role in determining drug delivery routes. Strategies have been devised to modify tropisms, aiming to reduce peripheral tropism while enhancing CNS tropism.^41^ Various AAV serotypes exhibit distinct tropisms toward different cell types; for instance, AAV9 shows affinity for astrocytes and neurons, while efforts have been made to develop AAV variants with tropism for oligodendrocytes,^104^ potentially targeting white matter injuries associated with oligodendrocyte degeneration. However, it is essential to consider that AAV tropism can vary between species and even across different ages within a species, underscoring the need for caution when interpreting data across diverse experimental models. AAV’s limited cargo capacity, typically around 4.7 kb, poses challenges, particularly for diseases like DS with mutations in genes such as *SCN1A* that require larger payloads. Strategies to address payload limitations include delivering smaller regulatory genes to enhance the expression of target genes, employing truncated but functional cassette genes, and exploring hybrid or dual AAV constructs.^143^ These approaches hold promise for overcoming cargo constraints and improving therapeutic outcomes in AAV-based gene therapy.

## Immune response

The immune response represents a significant concern following AAV administration, potentially arising from systemic, CSF, or local parenchymal injections, despite the brain traditionally being viewed as an immune-privileged site. Immune reactions can be elicited by various components, including the AAV capsid, transgene cassette, and encoded proteins.^58^ Moreover, It is crucial to note the interspecies variations when translating preclinical data to clinical studies, particularly regarding differences in immune systems and their development across different species.^144^ Consequently, diverse strategies have been devised to mitigate immune responses post-AAV injection. These approaches encompass the use of immunosuppressants such as corticosteroids and rapamycin administered before, during, and after AAV administration, as well as the engineering of AAV capsids to evade immune detection.^58^ Additional tactics involve depleting unmethylated cytosine-phosphate-guanine motifs to prevent recognition by Toll-like receptor 9 in the transgene capsid^145^ or modifying AAV surfaces to evade immune recognition.^146^ Therefore, comprehensive consideration of these factors is imperative for optimizing the safety and efficacy of AAV-based gene therapies.

## Transfer from rare disease to common disease

In addition to their application in monogenetic diseases, AAV-based gene therapies hold promise for addressing common and complex disorders through various strategies. These include the augmentation of neurotrophic factors such as brain-derived neurotrophic factor and nerve growth factor (NGF), anti-inflammatory cytokines like interleukin-10, and antioxidants such as superoxide dismutase. For instance, in Parkinson’s disease, AAV2-*GDNF* is intraputamenously delivered to patients (NCT04167540), while glutamic acid decarboxylase is delivered via AAV into the subthalamic nuclei. Similarly, in Alzheimer’s disease, AAV2 is employed to deliver *NGF* into the nucleus basalis of Meynert.^147^ These approaches exemplify the potential of AAV-based gene therapies in targeting diverse pathogenic mechanisms underlying complex diseases, offering avenues for therapeutic intervention beyond monogenetic disorders.

The convergence of mutation genes implicated in both monogenetic and complex neurodegenerative diseases presents a compelling opportunity for the development of AAV-based gene therapies that can address both rare and common disorders. This dual-purpose potential incentivizes the pharmaceutical industry to invest in such therapies, as they hold promise for not only treating rare diseases but also achieving commercial success in tackling more prevalent conditions. For instance, AAV-*GBA1*, designed to address *GBA1* mutations in type II Gaucher disease, can also be repurposed to target Parkinson’s disease, given that *GBA-1* mutations represent a common genetic risk factor for both conditions.^148^^,^^149^ Similarly, AAV-*AADC* therapy, approved by the FDA for AADC deficiency, has also been explored for Parkinson’s disease to restore dopamine synthesis, as evidenced by completed clinical trials^150^^,^^151^ and an ongoing clinical trial (NCT03562494). Another strategic approach fostering industry interest in AAV-based gene therapies involves delivering genes critical for a group of diseases, rather than focusing on a single rare disease. For example, AAV-mediated delivery of opsins holds the potential for addressing various retinal degenerative diseases, offering a versatile therapeutic platform capable of addressing multiple rare and common disorders simultaneously.^152^ These multifaceted strategies demonstrate the versatility and commercial appeal of AAV-based gene therapies in tackling diverse disease landscapes.

## Conclusions

In addition to the factors previously discussed, several other considerations can significantly impact the success rate of translating AAV-based gene therapies from preclinical research to clinical application. These factors include dosing regimens, timing of administration, and the selection of appropriate animal models for preclinical testing. Furthermore, many pediatric monogenetic diseases characterized by neurological pathologies have yet to progress to clinical trials due to various obstacles, such as primary mitochondrial disorders, fragile X syndrome, Angelman syndrome, SLC13A5 deficiency disorder, SLC6A1-related disorder, tuberous sclerosis, and CDKL5 deficiency disorder. Overcoming these barriers, including refining dosing protocols, optimizing treatment timing, and enhancing preclinical modeling, holds the potential to pave the way for the development of AAV-based gene therapies targeting a broader spectrum of diseases, encompassing both rare and common disorders. As advancements continue to address these technical challenges, the horizon for AAV-based gene therapies appears promising, offering hope for addressing an array of unmet medical needs across diverse disease areas.

## Acknowledgments

The funding was provided by 10.13039/501100004359Vetenskapsrådet, Sweden, grant no. 2022-01019, and the 10.13039/501100001809National Natural Science Foundation of China, grant no. U21A20347.

## Author contributions

L.Z. drafted the manuscript, and the other authors revised the manuscript. All authors commented on or edited the manuscript. All authors read and approved the final manuscript.

## Declaration of interests

The authors declare no competing interests.
